# Complete genomes of Australian *Bradyrhizobium* genospecies A and P representative strains

**DOI:** 10.1128/mra.00490-25

**Published:** 2025-06-18

**Authors:** Christine Oger-Desfeux, Jérôme Briolay, Philippe M. Oger, Bénédicte Lafay

**Affiliations:** 1 FR3728 BioEEnViS, PRABI-AMSB, Univ Lyon, Universite Claude Bernard Lyon1 27098, Villeurbanne, Auvergne-Rhône-Alpes, France; 2 FR3728 BioEEnViS, CNRS, DTAMB, Univ Lyon, Universite Claude Bernard Lyon1 27098, Villeurbanne, Auvergne-Rhône-Alpes, France; 3 CNRS, UMR5240, Univ Lyon, INSA Lyon, Universite Claude Bernard Lyon1 27027, Villeurbanne, Auvergne-Rhône-Alpes, France; 4 CNRS UMR5558, Laboratoire de Biométrie et Biologie Évolutive, Univ Lyon, Universite Claude Bernard Lyon1 27098, Villeurbanne, Auvergne-Rhône-Alpes, France; DOE Joint Genome Institute, Berkeley, California, USA

**Keywords:** *Bradyrhizobium*, Australia, genome

## Abstract

We report the complete genome sequences of *Bradyrhizobium* strains BDV5028 and BDV5111, representatives of *Bradyrhizobium* genospecies A and P, respectively, that symbiotically associate with Australian native legume hosts. The complete genome sequences provide genetic references for these Australian genospecies.

## ANNOUNCEMENT

*Bradyrhizobium* strains BDV5028 and BDV5111 are the representative strains of Australian *Bradyrhizobium* genospecies A and P, respectively. These genospecies, characterized using small subunit ribosomal RNA gene phylogeny, were the most frequently encountered rhizobial lineages, both across host species and sampling sites, in a survey of rhizobia associated with native shrubby legumes in south-eastern Australia in 1994–1995 (1). *Bradyrhizobium* genospecies A was found to be dominant overall across Papilionoideae and Mimosoideae hosts in temperate southeastern Australia (1–3), while *Bradyrhizobium* genospecies P occurred in different climatic and edaphic conditions across the whole Australian continent and exhibited a broad host range encompassing all three Fabaceae subfamilies (1–4). Strains BDV5028 and BDV5111 were isolated in January 1995 in New South Wales. BDV5028 was isolated from a root nodule of *Bossiaea ensata* (Fabaceae: Faboideae: Bossiaeeae) collected at Tianjara Falls, while BDV5028 was isolated from a root nodule of *Daviesia leptophylla* (Fabaceae: Faboideae: Mirbelieae) collected at Mundoonen Range.

Both strains were grown from lyophilized stocks in 30 mL of yeast extract mannitol broth (5) at 25°C and 200 rpm for 5 days. Genomic DNA was prepared as described by successive phenol-chloroform extractions (6). DNA quantification and quality control were assessed using a Nanodrop spectrophotometer, a Qubit 4 fluorometer, and agarose gel electrophoresis. Long reads were obtained using ONT MinION flowcell FLO-MIN112 from libraries prepared with the SQK-RAD004 kit using 1 µg of DNA and 20 second tagmentation. Basecalling was performed using Guppy version 6.4.8. Sequence quality was assessed using NanoPlot-1.42.0 (7). ONT long reads were cleaned from sequence adaptors using Porechop version 0.2.4 (8) and filtered for length (>1,000 bp) and quality (>10) using Chopper version 0.8.0 (7). Filtered reads were assembled using Flye version 2.9.4 (9) (options -nano-hq -scaffold -iterations 4) into single circular chromosome sequences, which were rotated to start at *dnaA* using Dnaapler-1.1.0 (10). Reads were aligned to these rotated sequences using Minimap2-2.28 (11) and Samtools-1.19 (12) prior to polishing with Medaka-2.0.1 (13). The assembly was carefully inspected by visualizing the alignment of reads onto the final assembly using Minimap2 version 2.28, Samtools-1.19, and IGV 2.16.2 (14). Completeness and redundancy of the final assemblies were checked using BUSCO version 5.8.2 (15) with the nitrobacteraceae_odb12 database. All softwares were run with default parameters unless otherwise specified. The assembled genomes were automatically annotated by the National Center for Biotechnology Information Prokaryotic Genome Annotation Pipeline version 6.9 (16). Genomic characteristics and BUSCO scores are provided in Table 1.

**TABLE 1:** Genomic features of *Bradyrhizobium* strains BDV5028 and BDV5111

	BDV5028	BDV5111
Genome GenBank accession	GCA_049949275	GCA_049949265
Filtered reads	377.6 Mb	1.27 Gb
Reads *N*_50_	8,669	5,181
Coverage	45×	129×
BUSCO score: complete (single copy, duplicated)	99.2% (98.8%, 0.4%)	99.5% (98.1%, 1.3%)
Chromosome length (bp)	8,387,507	9,941,418
Average GC content (%)	62.95	62.46
Genes	7,850	9,554
Protein-coding genes	7,617	9,085
tRNAs	52	53
5S rRNA gene	1	1
16S rRNA gene	1	1
23S rRNA gene	1	1
Pseudogenes	174	409

## Data Availability

The genome sequences of *Bradyrhizobium* genosp. A strain BDV5028 and *Bradyrhizobium* genosp. P strain BDV5111 are available in NCBI under accession numbers CP188196 and CP188195, and their raw sequence reads from SRA numbers SRX28460566 and SRX28460567 under the BioProject number PRJNA662585 and BioSample numbers SAMN48030122 and SAMN48030123, respectively. The strains are available on request from the corresponding author.
